# A grounded theory study in blended learning of the facilitation of role transition from compliant learners to collaborative partners regarding non-verbal behavior

**DOI:** 10.3389/fpsyg.2026.1871082

**Published:** 2026-08-19

**Authors:** Huali Fan, Shuai Shao, Shengwu Pan, Yuan Wang

**Affiliations:** 1 School of Modern Media, Xinjiang Teacher’s College, Urumqi, Xinjiang, China; 2 School of Educational Science, Xinjiang Normal University, Urumqi, Xinjiang, China; 3 School of Mechanical and Electrical Engineering, Xinjiang Institute of Engineering, Urumqi, Xinjiang, China

**Keywords:** collaborative learning, grounded theory, identity transition, nonverbal behavior, online collaboration, role-shifting

## Abstract

**Background:**

In blended learning environments, college students often remain silent in face-to-face classrooms yet actively engage in online collaborative settings. This gap reflects a shift in learner identity from “compliant learners” to “collaborative partners.” However, little is known about how non-verbal behaviors drive this identity transition.

**Methods:**

This study employed a grounded theory approach. Data were collected through in-depth interviews with 12 college students, supplemented by WeChat chat logs and collaborative document revision histories, and analyzed using three-level coding.

**Results:**

The findings reveal three categories of non-verbal behaviors that facilitate identity transition: emotional expression, relational coordination, and task support. These behaviors support a progressive mechanism comprising three stages: emotional resonance lays the foundation, boundary dissolution breaks existing frameworks, and relational empowerment affirms the new collaborative identity.

**Conclusion:**

Non-verbal behaviors facilitate a dynamic identity shift from compliant learners to collaborative partners. The study provides a theoretical model explaining micro-level mechanisms and offers practical insights for designing online collaborative activities.

## 1 Introduction

Blended learning, particularly models like the flipped classroom, is now a mainstream approach in higher education that combines traditional classroom learning with after-class learning (Böhm-Fischer and Beyer, 2024; McCarthy and Palmer, 2023). Early research focused on whether computer-mediated communication would lead to more egalitarian interactions (Tilak et al., 2023; Wang et al., 2024). More recently, however, attention has shifted toward how learners actively construct their digital identities within online spaces (Guraya et al., 2025; Wong, 2022). Despite technology providing ample support for collaborative learning, “classroom silence” among students remains a common phenomenon. In teacher-led classrooms, many college students remain silent and participate through non-verbal behaviors such as taking notes or photographing slides (Briffett-Aktaş and Ying, 2025; Chen J. et al., 2025; Le, 2024). However, when these same students move to social media platforms like WeChat or QQ outside of class, their behavior changes noticeably (Guo, 2022; Huang et al., 2023). They interact freely and participate actively (Shao and Zhang, 2025; Xing, 2025). This contrast suggests that learning behaviors and identities are reshaped as the teaching environment changes (Hod et al., 2024; Szynkiewicz, 2026). In this paper, a compliant learner is defined as a student who responds to institutional demands and completes assigned tasks in classroom settings (Jackson and Zmuda, 2014; McDonald et al., 2026); a collaborative partner is a learner who participates in post-class learning with a strong emphasis on joint creation, active decisions, and mutual support.

Non-verbal behavior serves as a rich and critical source of interactive cues in both face-to-face and online collaboration (Paneth et al., 2024). In traditional social interactions, non-verbal behaviors include eye contact, head movements, gestures, facial expressions, and body posture. In online collaborative learning, non-verbal behaviors take digital forms (Osei Tutu et al., 2025). Drawing on existing research, four categories of digital non-verbal behavior can be identified. These involve embodied behaviors in synchronous collaboration (Henry et al., 2025; Jeitziner et al., 2024), digital traces in collaborative editing (Han and Isaacs, 2025; Schubert et al., 2025), symbolic social presence in asynchronous discussions (Demir et al., 2025; Suen and Hung, 2025), and behaviors reflecting collaborative norms and relationship adjustments (Glikson and Riordan, 2024; Magni et al., 2025). In digital learning environments, using technological tools such as emojis and design techniques to convey a sense of presence and engagement helps bridge the psychological distance with learners (Gordon and Cole, 2026). Liu et al. (2021) found that students’ emotional interaction quality in online collaborative learning was of moderate quality on the whole, and positive interaction was much more common than negative interaction. In addition, emotional interaction seems to promote cognitive interaction (Uştuk et al., 2025). Paneth et al. (2024) demonstrated that specific non-verbal behaviors, such as head nodding and eye contact, are significantly associated with high-quality engagement in face-to-face collaborative learning. Jeitziner et al. (2024) further found that similar non-verbal behaviors can serve as meaningful indicators of collaborative group engagement in virtual synchronous settings. Indeed, recent research on digital non-verbal cues has shown specific patterns. The use of emojis can effectively predict learners’ engagement across both task and relational dimensions (Ferdinand, 2024; Graftdijk et al., 2025). These studies confirm the multidimensionality and measurability of online non-verbal behavior, but most still treat it as a static indicator of emotion or engagement, with few exploring its role in dynamic role adaptation.

Research on identity in online collaboration has reached a consensus that learner identities are dynamic rather than fixed (Papadopoulou and Welland, 2026). Individuals’ roles in online collaboration evolve dynamically (Coetzee et al., 2025; Elmoazen et al., 2025). Roles are influenced by awareness of participation, collaborative interaction, and balanced development. The core changes experienced by learners go beyond the linguistic level, encompassing transformations in self-expression behaviors, emotional attitudes, role cognition, and cross-cultural identity imagination (Lu, 2025; Zhang and Gonzales, 2025). Although traditional non-verbal cues are reduced in virtual environments, digital non-verbal cues (such as emojis, GIFs, and punctuation marks) have become new carriers of signals (Abjalova et al., 2025; Glikson and Riordan, 2024). The success of role transitions within virtual teams largely depends on whether members can promptly pick up on subtle signals during interactions (Gasson et al., 2006; Glikson and Riordan, 2024). Research has found that the use of these digital cues allows observers to perceive stronger emotional intensity, which in turn enhances the sense of closeness among team members and their motivation to join the team (Glikson and Riordan, 2024).

However, existing research primarily focuses on traditional classroom settings and has not explored the micro-mechanisms of non-verbal behavior in emotional transmission and role construction, nor has it sufficiently extended to online collaboration. These involve how learners actively negotiate identity transitions via non-verbal behaviors during cross-contextual shifts (Dixson et al., 2017; Frenkel et al., 2024; Zheng et al., 2024). This gap makes it difficult to address the unique characteristics of online non-verbal behavior. To address this gap, this study employs a grounded theory approach to explore how college students’ non-verbal behaviors facilitate identity transition in blended learning. Two research questions guide this study: (1) In blended learning, which non-verbal behaviors prompt students to shift from compliant learners to collaborative partners? (2) What underlying mechanisms support this transition process? We hypothesize that non-verbal behaviors underpin a progressive identity transition from “compliant learner” to “collaborative partner”.

## 2 Materials and methods

### 2.1 Research methodology

This study employs the Grounded Theory approach, a qualitative methodology first articulated by Glaser and Strauss (1967) and subsequently refined by Strauss (1987) and Charmaz (2006). Grounded theory emphasizes the systematic collection of raw data and stepwise coding of data to extract concepts and categories, ultimately developing a theory to explain a particular phenomenon or problem (Chen, 1999; Jia and Heng, 2020; Ruppel and Mey, 2015; Turner and Astin, 2021). Recent scholarship continues to affirm the relevance of grounded theory for investigating process-oriented phenomena in technology-mediated learning environments, as its inductive, iterative approach is well-suited for examining emergent interaction patterns in digital educational contexts (Ge and Liu, 2026; Ma, 2025; Rahimi and Khatooni, 2024). This method suits process- and mechanism-oriented research and aligns with this study’s aim to reveal the mechanisms underlying non-verbal behavior’s role in facilitating identity shifts. The research follows the three steps of classic Grounded Theory: open coding, axial coding, and selective coding (Strauss, 1987).

### 2.2 Participants

This study employed a purposive sampling strategy (Chen, 2000; Jiang and Guo, 2025; Turner and Astin, 2021; Wu et al., 2016). The study was conducted at a university, located in Urumqi, Xinjiang, China. Participants were recruited from Computer Science, Educational Technology, and Chinese Language and Literature. Inclusion criteria were as follows: (1) participants had completed at least one online collaborative course lasting 8 weeks or longer, ensuring sufficient exposure to sustained collaborative interactions; (2) participants were able to recall and articulate the non-verbal interactions that occurred during collaboration with adequate detail; (3) participants had experienced a perceived role transition from passive classroom participation to active online collaboration; and (4) participants were willing to share WeChat chat logs and collaborative document revision histories from their online collaboration. Exclusion criteria included: (1) unwillingness to participate in the study or share data; (2) inability to articulate experiences clearly; and (3) insufficient understanding of the interview topics, relevant concepts, and interview questions, which would compromise the depth and richness of data. All inclusion criteria and exclusion criteria were reviewed by experts in qualitative research methodology (Chen W. et al., 2025).

The recruitment period of this study started on 01/03/2026 and ended on 25/04/2026. Data collection and analysis were conducted during this period, and a total of 12 college students participated in the study. Table 1 presents the demographic and academic characteristics of the participants. All participants had completed at least one collaborative course lasting 8–12 weeks, ensuring sufficient exposure to sustained online collaborative learning experiences. The sample size was determined based on the principle of theoretical saturation. Data were organized immediately after each interview. Interviews were stopped when respondents’ answers began to repeat, with no new information added.

**TABLE 1:** Interview participants.

ID	Major	Collaborative course	Collaboration duration	Gender	Year of study
I1	Computer Science	Python	12 weeks	Male	Freshman
I2	Educational Technology	Educational Technology Research Methods	8 weeks	Female	Sophomore
I3	Educational Technology	Educational Technology Research Methods	8 weeks	Male	Sophomore
I4	Chinese Language and Literature	Traditional Chinese Culture	8 weeks	Female	Freshman
I5	Chinese Language and Literature	Traditional Chinese Culture	10 weeks	Female	Sophomore
I6	Computer Science	Python	10 weeks	Male	Freshman
I7	Computer Science	Python	12 weeks	Male	Freshman
I8	Computer Science	Python	8 weeks	Female	Sophomore
I9	Chinese Language and Literature	Traditional Chinese Culture	10 weeks	Female	Sophomore
I10	Educational Technology	Educational Technology Research Methods	8 weeks	Male	Sophomore
I11	Computer Science	Python	8 weeks	Female	Freshman
I12	Educational Technology	Educational Technology Research Methods	10 weeks	Female	Sophomore

### 2.3 Ethical issues

This study’s protocol was approved by the Ethics Committee of Xinjiang Teacher’s College (approval number: “XJEDU2026LLSC006”). Participants were informed of the research’s purpose before the interview. All participants have signed the informed consent form. We told the participants that the interview data would be used only for academic research and could be kept confidential. The interview could be conducted after the participants had signed the Informed Consent Form. Sensitive information such as names, class, and school was coded with a code (such as “I1,” “I2”) to protect their identity. The records and documents were encrypted and can only be accessed by the researcher. The participants were given preliminary findings of the study to ensure the right to be informed. Once requested, content needs to be changed or removed, the data can be adjusted promptly.

### 2.4 Data collection

#### 2.4.1 Semi-structured interviews

The study employed semi-structured interviews. The sequence was as follows: The interview guide was first designed using evidence-based principles, and a draft was created from a literature review. Next, pilot interviews were conducted to validate and refine the draft. Then, domain experts reviewed the guide to ensure validity. The interview guidelines were further modified by the research group following the first three interviews, depending on the feedback (see Appendix A). Interviewers were trained in grounded theory methods and proficient in probing techniques, enabling participants to articulate their experiences and have good probing skills, enabling participants to articulate their experiences and feelings and providing optimal descriptions to extract information (Jiang and Guo, 2025). Interviews were conducted online or face-to-face. Before starting the interview, research objectives, concepts, and themes were explained to the participants, and participant consent was obtained. Each participant was interviewed in one to two interviews. Each session lasted 30–50 min. During the interviews, the interviewer remained theoretically sensitive and probed further into the concepts participants brought up. After the interviews, audio recordings were transcribed. This process yielded about 80,000 characters. Valid information was manually screened and verified with the participants. The transcripts were labeled I1-I12 (where “I” represents the interviewee).

#### 2.4.2 Text data collection

The collected text data is organized into three categories. First, we collected WeChat chat logs from group collaborations, covering the early, middle, and late stages. We extracted non-verbal information such as emojis, interjections, and response rhythms, categorizing emojis by type, retrieving interjections using keywords like “ya,” “la,” “yo,” and “∼,” and calculating response rhythms based on intervals between timestamps. Second, course forum posts containing interactive content from group discussions were analyzed, with a focus on non-verbal behaviors such as likes, emoji comments, and reactions. Third, revision logs of collaborative documents, including revision histories in Tencent Docs, were reviewed to track encouraging annotations and the rhythm of co-editing, along with other non-verbal cues. We selected textual data for its direct relevance to non-verbal behaviors, excluding content such as pure task assignments or notifications. Each participant provided three sets of textual data, resulting in 36 data sets labeled D1-D36.

### 2.5 Data analysis

NVivo 12 software was used to facilitate data management and coding throughout the analysis process. The software enabled systematic organization of interview transcripts and text data, supported the creation of hierarchical coding structures, and allowed for efficient retrieval of coded segments across multiple data sources. Data analysis followed the iterative, comparative, and inductive process of grounded theory (Glaser and Strauss, 1967). Analysis began immediately after the first round of interviews and proceeded in parallel with data collection. Ten interview transcripts were randomly selected for qualitative coding using NVivo 12 software, while the remaining two transcripts were used to test for theoretical saturation (Rahimi and Khatooni, 2024).

#### 2.5.1 Open coding

Open coding begins with a sentence-by-sentence analysis to conceptualize and categorize the raw data (Chen, 2000). The coding was conducted jointly by two graduate students, both trained in grounded theory coding. Each coded the data independently, using a back-to-back approach. To ensure consistency, a verification process was conducted after each coding level. The initial inter-coder reliability was 87.3%, exceeding the generally accepted 80% standard in qualitative research (Miles and Huberman, 1994). Disagreements that arose during initial coding were resolved through discussion and consultation. When consensus could not be reached, a third researcher was consulted to make the final decision. During categorization, any concept not related to the research question or occurring fewer than 3 times was excluded. Eventually, 25 initial categories emerged (see Table 2). For example, A1 and A2 were derived from participants’ descriptions of emoji use (I5, I3), relating to the functions of emojis in expressing collaborative willingness and creating an interactive atmosphere. A3 was derived from WeChat logs where emojis conveyed empathy (D12).

**TABLE 2:** Example process of transformation from raw data to concepts.

Primary source material	Conceptualization	Categorization
When I first sent out the task assignment sheet, I used the emoji “[clasped fists].” My partner replied with “[thumbs up]” and said, “Let’s work together to tell this story well∼.” Suddenly, it didn’t feel like I was just “going through the motions.” (I5 - Chinese Language and Literature).	Emotions convey a willingness to collaborate	A1 The expression of identity through emojis
At first, I would just send “Task complete,” and my partner would reply with “Got it.” Later, I added “[smile],” and he started sending “[thumbs up].” Gradually, he became more willing to share his thoughts. (I3 - Educational Technology)	Emoticons help create an interactive atmosphere	A2 The icebreaker effect of emojis
Someone in the group chat posted a “[bitter]” emoji and said, “This part is so hard.” Several people replied with “[hug]” and “[cheer]” emojis, and I instantly felt like I wasn’t fighting this battle alone. (D12 - WeChat chat log).	Expressing empathy and support through facial expressions	A3 The emotional comfort of emojis
More cohesive teams engaged in substantially more synchronous editing, while less cohesive teams engaged in notably less. (D25 - Tencent Docs Edit History)	Link performance to the duration of synchronization	A24 Synchronized editing and collaborative efficiency
Sometimes I write the outline first, and my partner fills in the details a few hours later. Even if we’re not working at the same time, it feels like we’re passing the baton, like in a relay race. (I12 - Educational Technology)	The asynchronous relay creates a sense of continuity	A25 Collaborative editing in relays

#### 2.5.2 Axis coding

Axis coding involves further analyzing and organizing open coding to establish relationships among the initial categories and to abstract higher-level main categories (Wu et al., 2016). By comparing and synthesizing the semantic relationships and attribute correlations among the 25 initial categories, six main categories are identified, as shown in Table 3. B1 addresses emojis’ roles in conveying empathy, breaking the ice, providing emotional comfort, affirmation, and mood regulation. B2 addresses particles’ roles in expressing warmth, de-formalizing communication, conveying inclusivity, and guiding knowledge sharing. B3 addresses response immediacy, delayed response effects, response speed and group effectiveness, and group synchronization. B4 addresses interaction continuity, sense of closeness, dynamic adaptation, and compensation mechanisms. B5 addresses suggestive tones, encouraging expressions, inquisitive questions, and community orientation in comments. B6 addresses synergy in editing rhythm, perception of synchronization, synchronized editing and collaborative efficiency, and relay-style collaboration in editing.

**TABLE 3:** Results of axial coding.

Main category	Corresponding to the initial category	The essence of relationships
B1 Emotional expression through emojis	A1 Conveying empathy through emojis A2 The ice-breaking effect of emojis A3 Emotional comfort provided by emojis A4 The affirmative function of emojis A5 Mood regulation through emojis	Learners use emojis to convey acceptance, empathy, and support, help set the tone of the interaction, and establish an initial emotional connection.
B2 The role of particles in conveying intimacy	A6 Expressing warmth through interjections A7 De-formalizing communication with interjections A8 Expressing inclusivity through interjections A9 Guiding knowledge sharing with interjections	Learners use colloquial particles to soften the authoritative tone of the task language, bridge the psychological gap, and foster an inclusive atmosphere.
B3 Synchronization with the rhythm	A10 Immediacy of responses A11 Delayed response effects A12 Response speed and group effectiveness A13 Group synchronization in responses	Learners establish a synchronized rhythm and pace of interaction, transforming “task time” into “partner time” and fostering a sense of trust.
B4 Adaptability of interaction frequency	A14 Interaction continuity A15 Sense of closeness through interaction frequency A16 Dynamic adaptation of interaction frequency A17 Interaction compensation mechanisms	Learners dynamically adjust their interaction frequency as the task progresses, thereby maintaining a sense of “continuous presence” and reinforcing role stability.
B5 The tone of the document comments	A18 Suggestive tone in comments A19 Encouraging expressions in comments A20 Inquisitive questions in comments A21 Community orientation in comments	Learners provide feedback in a suggestive or encouraging tone, empowering the recipient through positive reinforcement.
B6 The rhythm of collaborative editing	A22 Synergy in editing rhythm A23 Perception of synchronization in editing rhythm A24 Synchronized editing and collaborative efficiency A25 Relay-style collaboration in editing	Learners establish a collaborative rhythm through synchronous or relay-style editing, fostering a team experience of “co-creation.”

#### 2.5.3 Selective coding

By analyzing and organizing the relationships among the various main categories, we can identify the core categories. The relationships between the main categories and the core categories are shown in Table 4. The six main categories are grouped into three core dimensions, each playing a distinct role in the identity transition process. Emotional expression behaviors (C1) establish the foundation by generating emotional resonance; relationship coordination behaviors (C2) facilitate boundary dissolution by shifting interaction patterns from directive to responsive; and task support behaviors (C3) reinforce the new identity through relational empowerment. Together, these three dimensions form a progressive mechanism that explains how identity transition unfolds.

**TABLE 4:** The relationship between main categories and core categories.

Dimension	Main Category	Key Role	Relationships Among Core Categories
C1 Non-verbal behaviors related to emotional expression	B1, B2	Emotional resonance	Convey goodwill and recognition through emojis and interjections, evoke emotional resonance, break down identity barriers, and lay the psychological groundwork for identity transition.
C2 Non-verbal behaviors related to relationship coordination	B3, B4	Boundary dissolution	By synchronizing responses to the rhythm and adjusting the frequency of interaction, we dissolve the supervisor-supervisee framework of classroom rules and loosen the existing role definitions.
C3 Non-verbal behaviors in task collaboration	B5, B6	Empowering relationships	Through encouraging feedback and real-time collaboration, learners are empowered with a voice and decision-making authority, reinforcing their identity as collaborators.

From Table 4, non-verbal behaviors directly convey emotions and intentions, serving as cues to break free from situational roles. Learners express emotions and set the mood non-verbally. This helps form emotional connections and lays the foundation for identity transformation. Then, when learners synchronize their responses and adjust the frequency of their interactions, they move beyond one-way classroom rules. Through this progression, the “supervisor-supervisee” boundary dissolves, further loosening the role of the “compliant learner.” Last, as learners use suggestive annotations and engage in collaborative editing rhythms, they strengthen participation and affirm their identity as equal collaborative partners. In this way, proactive non-verbal behavior in online collaborative learning may contribute to a shift from a passive compliant learner to a collaborative partner. This transformation unfolds through a three-stage process: emotional resonance, boundary dissolution, and relational empowerment.

#### 2.5.4 Theoretical saturation test

Theoretical saturation is a crucial test for assessing the adequacy of the sample in qualitative research (Xie and Chen, 2021). Theoretical saturation was assessed following the principle of constant comparison (Glaser and Strauss, 1967). After coding the 10th interview transcript, no additional initial categories emerged. However, to ascertain saturation at a higher analytical level, we further examined whether the properties, dimensional variations, and relational patterns among the six main categories and three core dimensions had been sufficiently developed. The two subsequent interviews were coded and compared against the existing coding framework. The results confirmed that: (1) no new initial categories were identified; (2) the properties of each main category (e.g., types of emojis, response intervals, annotation tones) exhibited no novel variations; (3) the directional relationships among the three core dimensions were consistently supported without requiring additional relational postulates. Consequently, the two additional interviews served to validate rather than extend the emergent theoretical model (Turner and Astin, 2021). Thus, the theoretical model constructed in this study has passed the theoretical saturation test.

#### 2.5.5 Cross-source validation

To mitigate the inherent limitations of retrospective interview data, this study employed a cross-source triangulation strategy. We validated data by cross-referencing interviews and text materials. Interview themes were compared with patterns identified in WeChat chat logs, course forum records, and Tencent Docs revision histories (Patton, 2015). When discrepancies were identified between interview accounts and textual records, follow-up interviews with the relevant participants were conducted to clarify interpretations. This multi-source validation approach ensured that the findings were not solely dependent on retrospective interview accounts (Belgrave and Seide, 2019; Chen, 1999; Wu et al., 2016).

## 3 Results

**FIGURE 1:**
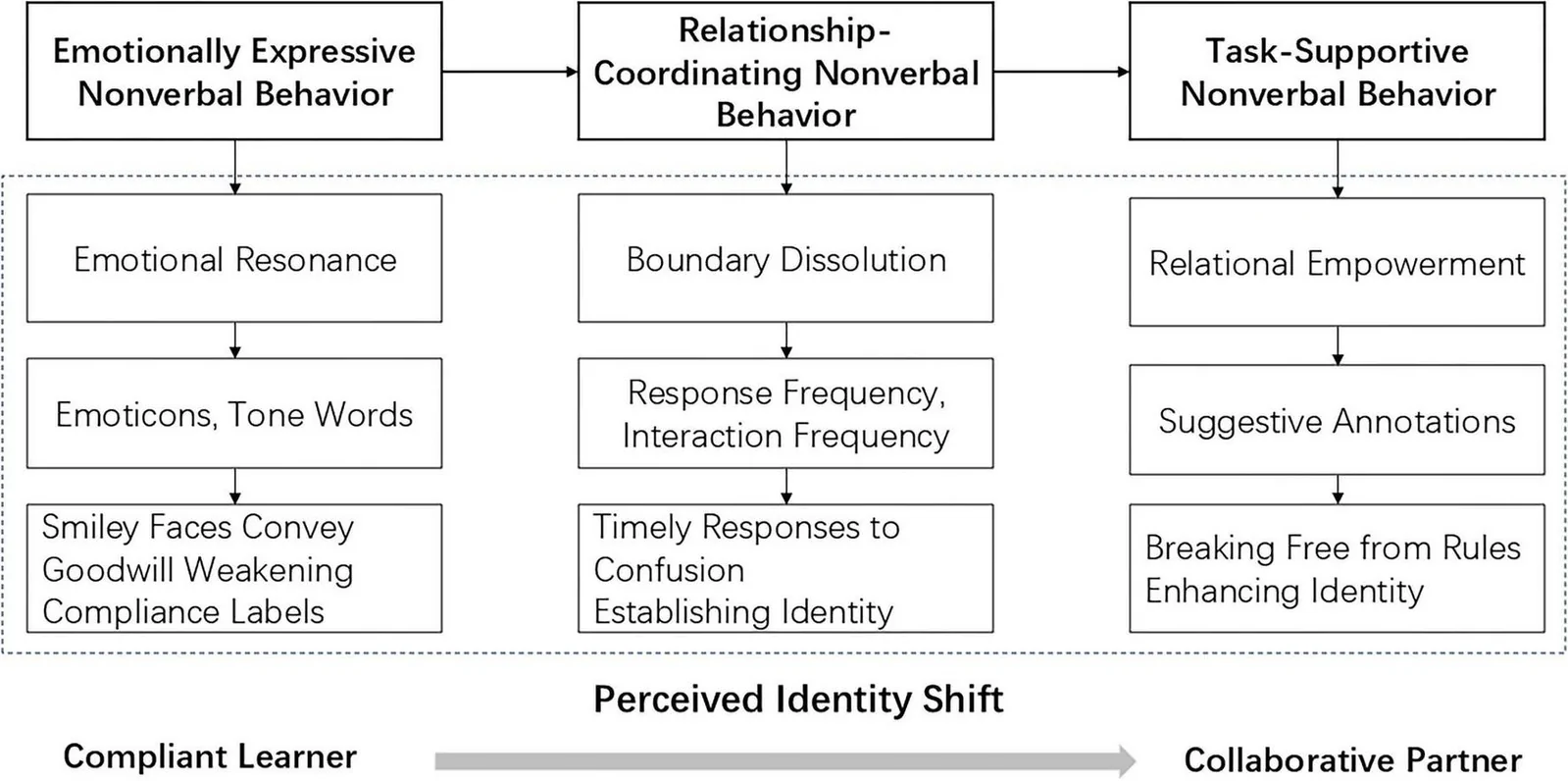
A model of non-verbal behaviors facilitating identity transitions in online collaborative learning. Source: Author-created.

Based on the analysis and coding of the interview and textual data, this section constructs a theoretical model (see Figure 1) to explain how non-verbal behaviors facilitate the identity transition from compliant learners to collaborative partners in online collaborative learning. The model clearly demonstrates that this identity transition is not driven by a single type of non-verbal behavior, but rather is a progressive process co-shaped by three categories of non-verbal behaviors: emotional expression, relationship coordination, and task support. The arrows indicate the temporal progression of the mechanism. Therefore, when examining how non-verbal behaviors promote identity transition in blended learning, it is essential to consider all three dimensions and their sequential interplay.

### 3.1 Non-verbal behaviors for emotional expression

Non-verbal behaviors for emotional expression refer to interactive methods that convey emotions through symbols such as emojis and interjections. The main role is to remove the invisible walls between students’ identities in class through emotion and then create a psychological base for others to develop a new feeling around the partners. There are two detailed aspects in this mechanism: one is to use emojis to convey emotions, and another is to use interjections to create a sense of intimacy.

Emojis are among the most direct tools for conveying emotions in online collaboration. In the interviews, several participants mentioned the “ice-breaking” effect of emoticons.

I5 said, “When I first sent the task assignment sheet, I used ‘[fist bump]’. My partner responded with ‘[thumbs up]’ and added ‘Let us tell this story well together∼’. Suddenly, I realized I was not just ‘going through the motions,’ but that someone was collaborating with me to complete the task.”

I3 offered another perspective: “Before, I just sent ‘Task completed,’ and my partner replied ‘Got it.’ As I began adding ‘[smile],’ he started replying with ‘[thumbs up].’ Gradually, we became more willing to share ideas, unlike the stiffness we felt before.”

Textual data supports this. D12 (WeChat chat logs) revealed a marked shift in interaction patterns over time. During the early phase (Weeks 1–2), messages were predominantly brief and task-oriented, with minimal use of affective markers. The chats consisted of “Got it” and “Completed as required.” By the middle phases (Weeks 3–6), the logs displayed a substantial increase in emoji usage. Content centered on “Let us refine this together” and “I’ll help you.” These examples show that emojis, as non-verbal carriers of emotion, break formulaic interaction patterns. They send intentions like “acknowledgment” and “support.” Emojis make people feel “seen” and “accompanied,” which fosters a sense of identity as learning partners. However, overusing or misusing emojis can weaken this identity. For example, I6 said: “Using emojis without substantive content feels unprofessional.”

The use of interjections fosters a sense of closeness and strengthens emotional connections by reducing the formality of language.

I9 described: “I posted ‘I have written a 3,000-word literature review’ and my friend said ‘Wow! That’s so detailed∼ Let’s look for some case studies to add together∼’ Because my friend used YA and ∼, I felt I was more than ‘classmates; the interjection gave this feeling of ‘best friends watching a show together.”’

I7 explained: “Previously, when I posted a coding problem, I just said ‘I am getting an error here.’ My partner said, ‘Check the variable name.’ Later, he began saying ‘The variable name might be wrong here∼ I have made that mistake before too.’ When my partner added ‘LA’ and ‘∼’, these interjections made me explain more details about the error, instead of just the fact, because the conversation felt more like support.”

These examples illustrate how de-formalizing particles transforms the task-oriented language of the classroom into everyday language. By reducing the formality of interactions, they facilitate a shift from rule-following learners to emotionally connected peers.

### 3.2 Non-verbal behaviors related to relationship coordination

Relationship-coordinating non-verbal behaviors are interactive strategies. They align with a partner’s needs by adjusting response rhythm and interaction frequency. These behaviors help dissolve boundaries set by classroom compliance rules. Specifically, they enable individuals to shift focus from procedural task completion to attentiveness toward partner needs. This promotes more genuine collaboration. This mechanism has two sub-dimensions: synchrony of response rhythms and adaptability of interaction frequency. Together, these help transform compliance-driven engagement into responsive collaboration.

The synchrony of responses directly influences shifts in identity perception.

I8 recalled, “Once, I messaged at 2 a.m.: ‘I am stuck in an infinite loop while debugging.’ Expecting to wait until the next day, I was surprised when my partner replied 15 min later: ‘I am working on this module too. Let us check the logs together,’ with a ‘[struggling]’ emoji. Instantly, I no longer felt like just a classmate completing an assignment, but a collaborative partner working intensively through the night.”

I10 said: “If I send a message and no one replies for a long time, I would feel like ‘they are just going through the motions’ and wouldn’t want to say much more; but if a partner responds quickly, I would proactively share more ideas, just like chatting with a friend.”

Textual data D21 (forum records) show a discernible difference in response patterns. Groups that exhibited more cohesive collaboration in their interviews tended to show notably shorter inter-reply intervals in their forum threads, characterized by rapid and continuous turn-taking. In contrast, groups that reported less cohesive collaboration displayed more sporadic and delayed response sequences.

I6 noted: “In groups with a good pace, I feel like we are on the same team. In slower groups, it is…we are just doing our own thing.” That synchrony of action and interaction is what connects the “task time” of the classroom to the “peer time” that happens in it. Responding to the immediate needs of those around us helps to move student work beyond the procedural compliance of “following instructions.” That shifts students’ identity from sole task-doers to drivers of action who respond to the needs of peers, the mechanism behind our study.

The responsiveness of interaction frequency consolidates the sense of partnership by creating a feeling of “continuous presence.” This consolidation links elastic communication directly to the feeling of ongoing collaboration.

I4 said, “Our group posts ‘Today’s Progress’ in the chat every day. It is not a mandatory requirement; rather, when a partner voluntarily says, ‘I revised the introduction today,’ I will reply, ‘I am working on the case study too.’ Gradually, this has become a daily habit, unlike before when we only spoke up at specific task milestones.”

Textual data D18 (WeChat chat logs) indicated a marked increase in daily interaction frequency over time. Groups with higher reported engagement showed substantially more frequent daily exchanges in the later collaboration stages compared to the earlier phases, reflecting a deepening pattern of continuous presence.

I2 noted: “Seeing my partner’s updates every day makes me feel like ‘they are walking this path with me,’ rather than ‘someone who disappears after completing a task.”’ This frequent “continuous presence” broke from the usual classroom, task-driven interaction. Partners felt they were always by each other’s side. Their identity shifted from “compliant learners” to long-term collaborative partners.

### 3.3 Task-supporting non-verbal behaviors

Task-supporting non-verbal behaviors are interactive actions that provide support in collaborative documents and project discussions, such as encouraging comments and synchronized editing. Their core function is to empower individuals by making them feel that their peers rely on them, thereby reinforcing their sense of identity as “collaborative partners.” This mechanism can be broken down into two sub-dimensions: encouraging comments in collaborative documents and synchronizing editing rhythms.

Encouraging comments on collaborative documents reinforce the sense of partnership through positive feedback.

I11 said, “Before, my partner’s comments were things like ‘Please fix line 5 of the code.’ Later, they changed to ‘Using a loop here would make it more concise—why not give it a try? I made that change myself before.’ I felt like he was ‘helping me’ rather than ‘pointing out mistakes,’ so I was willing to reach out to him proactively to discuss things, and I would even ask, ‘What do you think of this change?”’

I5 mentioned, “When my peer commented on my case study, saying, ‘This story is really heartfelt! Adding some data would make it more persuasive∼,’ I instantly felt that ‘my ideas have value,’ rather than just ‘filling the word count as required.”’

Textual data D30 (Tencent Docs revision history) shows that encouraging and suggestive annotations were relatively scarce in the early revision phases but became markedly more prevalent as collaboration progressed toward completion.

I3 said, “Seeing my partner’s encouragement, I will take on more tasks proactively, feeling that ‘we are working together to get things done,’ rather than just completing our assigned tasks individually.” These constructive comments basically convert evaluative classroom feedback into supportive feedback. By creating a sense of competence in people, these comments move their identity from evaluated learners to trusted partners.

The shared rhythm of collaborative editing enhances group identity by creating a real sense of presence and teamwork.

I7 said, “When one of us was writing a function and the other one was writing comments, I would get a message from my partner every 10 min asking, ‘I have completed this part,’ and I would reply, ‘I almost do too, let us test it.’ However, suddenly instead of ‘writing code separately,’ it seemed that we were constructing a house together.”

I9 said, “My partner and I would edit our paper online together. He was editing the introduction while I edited the conclusion. Sometimes he would send me a message saying, ‘I added a case study here,’ and I changed the conclusion’s phrasing. It was ‘two people writing an article together,’ not ‘two people writing separate parts.”’

The textual data D25 (Tencent Docs editing history) showed that high-cohesion groups engaged in substantially more synchronous editing episodes, characterized by overlapping revision timestamps and reciprocal cross-referencing between co-editors.

I12 said, “When we edit together, I feel ‘we are a team,’ not ‘individuals forced to come together for a task.”’ This new way of online learning by synchronous collaboration changes teachers’ and students’ ‘division and collaboration’ into ‘co-creation’, helping them perceive and feel each other’s interdependence, thereby making and clarifying their identity as collaborative partners.

### 3.4 Cross-source validation findings

By integrating interview narratives with naturally occurring digital traces, the triangulation procedure substantiated the interpretive validity of the findings.

The theme of emotional resonance found convergent support across sources. While interviewees (e.g., I5, I9) recalled specific moments when emojis or interjections reduced psychological distance, the corresponding WeChat logs (D12) displayed a noticeable clustering of empathy-related symbols during the same collaboration phases. Similarly, boundary dissolution was consistently observed in the course forum records (D21): groups characterized by rapid, alternating reply sequences in the textual data were precisely those whose members, in interviews, described their collaboration as “fluid” and “like chatting with friends.” For relational empowerment, the revision histories (D30) showed that participants who received more suggestive and affirmative annotations were also those who, during interviews, articulated a stronger sense of agency in decision-making. This alignment between spoken accounts and written traces suggests that the three-stage mechanism is not an analytical artifact but a pattern grounded in multiple layers of behavioral data.

A critical test of the theoretical model came from a deviant case (I1), who reported minimal personal use of emojis yet still felt a strong collaborative identity. Triangulating this outlier with textual records revealed that while I1’s own messages were sparse, his partner’s contributions were rich in encouraging non-verbal markers. This cross-source check indicated that the identity-facilitating effect does not depend on symmetrical reciprocity; unilateral positive non-verbal signaling, when perceived, can suffice to initiate role transition. This finding refined the theoretical model by highlighting perceived peer support as a boundary condition that can operate independently of the individual’s own expressive behavior.

## 4 Discussion

We can draw on emotional contagion theory (Hatfield et al., 1993; Suen and Hung, 2025), social presence theory (Demir et al., 2025; Short et al., 1976), and identity negotiation theory (Papadopoulou and Welland, 2026; Swann, 1987) to explain the research findings. In this framework, identity in online collaborative learning is understood not as a fixed individual attribute but as a dynamic process continuously constructed and negotiated through symbolic interactions. The progressive “emotional resonance–boundary dissolution–relational empowerment” mechanism revealed in this study provides a novel theoretical perspective for understanding this process. We discuss each of these three stages in turn, explaining how the relevant theoretical perspectives illuminate the specific mechanisms through which non-verbal behaviors facilitate identity transition.

### 4.1 Research findings

First, emotional resonance, as the initiating point of identity transformation, indicates that the emotional transmission function of non-verbal behavior is the psychological ground for identity. Chen W. et al. (2025) found in their asynchronous video-based learning study that instructors’ facial expressions and vocal charm affect learners’ emotional involvement through the “contagion of emotion” mechanism. The results of this study are in conversation with this research: In the context of peer collaboration, emojis and tone particles perform an analogous emotional contagion function. However, they serve not only to enhance emotional involvement, but rather transform “task executors” into “emotionally connected partners” by conveying emotional intent such as “identification” and “support.” This finding extends the application of the emotional contagion theory from the teacher-student interaction to peer collaboration. It empirically grounds Maehler and Hernández-Torrano (2025) assertion that identity transformation encompasses changes in emotional attitudes and self-expression behaviors.

Second, how the dissolution of role frameworks, as an essential process of identity negotiation, shows how non-verbal behavior extends beyond the role frameworks of the classroom setting. Shao and Zhang (2025) highlight that the root cause of classroom silence is the disruption of classroom ecosystem connections. In our research, we found that connection disruption can be restored by non-verbal behavior beyond the classroom. In particular, the synchrony of response rhythm and flexibility of interaction frequency change the task time of the classroom into partner time, and the interaction relationship from supervisor–supervisee to companionship–support of collaboration. Therefore, this finding may supplement the key concepts of the theory of social presence: the development of social presence is not only the result of “perceiving presence of others,” but a sense of “continuous presence” actively developed and fostered by non-verbal behavior, whose essence is the dissolution and reconstruction of role frameworks (Demir et al., 2025; Suen and Hung, 2025).

Third, relational empowerment is the last step in the process of identity transformation, in which non-verbal behavior gives learners a license to speak and decide. As found in our study, task-supportive non-verbal behaviors, such as annotating encouragements and simultaneous editing, are associated with learners’ sense that their peers depend on them and recognize them as competent collaborators, leading to substantial empowerment in collaborative relationships. This is consistent with identity negotiation theory (Maehler and Hernández-Torrano, 2025). Thus, non-verbal behaviors function not only as cues for assessing collaborative quality, as suggested by Paneth et al. (2024), but they also serve as a means of identity positioning through empowerment mechanisms. In contexts outside classroom, such non-verbal behaviors promote the emotional bonds, interactional coordination, and co-create knowledge, allowing them to move beyond rigid classroom roles and achieve dynamic identity transformation.

### 4.2 Theoretical contributions

This study shows that non-verbal behaviors in online collaboration can directly support identity transitions and makes three theoretical contributions. First, it develops a framework that clarifies how non-verbal behaviors facilitate identity transitions at a micro level (Glikson and Riordan, 2024; Uştuk et al., 2025). This framework serves as a practical tool for studying identity in online collaboration and creates a base for future quantitative research. Second, by highlighting the key role of non-verbal behavior in online identity building, this study broadens this theory’s use in digital education (Jeitziner et al., 2024; Uştuk et al., 2025). It helps learners perceive the quality of collaboration and construct their identity within collaborative relationships through emotional resonance, boundary dissolution, and relational empowerment. Third, by explaining the mechanisms of identity transition in online collaboration, this study clarifies the micro-processes where non-verbal behavior facilitates identity shifts (Glikson and Riordan, 2024). It maps the paths of identity transition from compliant learners to collaborative partners, providing qualitative evidence for online identity research in the sociology of education.

### 4.3 Practical implications

This study offers important insights into understanding student engagement in blended learning and provides concrete guidance for teachers in designing collaborative activities within such settings. First, teachers should promote emotional icebreakers through non-verbal behaviors that express emotions. At the beginning of collaborative activities, teachers can encourage students to use non-verbal cues, such as emojis and interjections to convey emotional intent, thereby reducing the formal barriers associated with classroom identities. For example, during the group formation phase, teachers can design a low-stakes ‘Emoji Self-Introduction’ activity to familiarize students with symbolic communication and establish emotional connections before task demands emerge (Ferdinand, 2024). Teachers may also encourage the use of supportive emojis during task assignments to foster an inclusive atmosphere (Graftdijk et al., 2025). Second, teachers should focus on the appropriateness of the rhythm and frequency of student interactions, guiding students to synchronize their response rhythms and cultivate the collaborative habit of providing timely feedback (Jeitziner et al., 2024); at the same time, they should allow groups to dynamically adjust interaction frequency based on the task stage to avoid interaction anxiety caused by an excessive emphasis on immediacy (Paneth et al., 2024). For instance, teachers can implement lightweight interaction mechanisms, such as daily progress check-ins, to help students maintain a sense of “continuous presence.” Finally, teachers can design collaborative tasks requiring synchronous editing and relay-style completion (Han and Isaacs, 2025). For example, teachers can assign tasks where students co-construct a shared document in real-time, with different group members taking responsibility for different sections while providing real-time feedback on each other’s contributions (Schubert et al., 2025). Teachers may also incorporate non-verbal behaviors, such as encouraging comments and inquiry-based questions, into grading criteria to reinforce the quality of peer feedback (Chen J. et al., 2025; Uştuk et al., 2025). This sends a clear signal that thoughtful, supportive non-verbal communication is valued as part of the collaborative learning process, thereby reinforcing students’ adoption of collaborative partner identities.

### 4.4 Limitations and future directions

This study has some limitations that should be addressed in subsequent studies.

First, while the sample size of 12 participants reached theoretical saturation, the small sample size limits the generalizability of findings. Some methodological scholars have recommended larger samples for grounded theory studies to enhance transferability (Glaser and Strauss, 1967; Turner and Astin, 2021). However, saturation in grounded theory is determined by data adequacy rather than predetermined sample size (Rahimi and Khatooni, 2024). Future research could expand the sample to include more participants from diverse backgrounds to enhance transferability.

Second, this study was conducted in Chinese higher education with Chinese platforms (WeChat and Tencent Docs). Communication norms, including the use of emojis, interjections (e.g., “∼” and “ya”), and response expectations, may differ significantly across cultures (Magni et al., 2025). This cultural specificity limits the generalizability of the findings to non-Chinese or cross-cultural collaborative learning settings. Future research should examine whether similar non-verbal mechanisms operate in diverse cultural and linguistic contexts.

Third, the collaborative courses in this study ranged from 8 to 12 weeks in duration, which may not capture the full trajectory of identity transformation in longer-term collaborations. Identity shifts may unfold differently over extended periods, potentially involving more complex negotiation processes (Ibarra, 1999; Maehler and Hernández-Torrano, 2025). Future studies could employ longitudinal designs to track how non-verbal behaviors and role transitions evolve over longer timeframes.

Fourth, this study focused exclusively on text-based non-verbal cues, including emojis, interjections, and editing rhythms. Multimodal behaviors, such as video-based facial expressions, gestures, and vocal intonation were not examined. This limits the applicability of the findings to synchronous video-mediated collaborative environments where richer multimodal cues are available (He et al., 2022; Paneth et al., 2024). Future research could extend the investigation to video-based collaboration platforms (e.g., Zoom, Microsoft Teams) to examine how multimodal non-verbal behaviors interact with text-based cues in shaping identity transitions (Hampel and Stickler, 2012; Mohammadi et al., 2025).

Fifth, the interview data relied on participants’ retrospective recall of their collaborative experiences, which may be subject to memory biases and reinterpretations (Rahimi and Khatooni, 2024; Schwarz, 1999). To mitigate this limitation, we triangulated interview data with contemporaneous text records (chat logs, forum posts, and editing histories), which served as temporal anchors for recall. However, retrospective bias cannot be fully eliminated. Future research could employ real-time data collection methods, such as experience sampling or continuous screen recording, to capture immediate perceptions of non-verbal interactions and reduce reliance on retrospective accounts (Bolger et al., 2002; Mosen et al., 2026).

Sixth, the sample was drawn from three disciplines within a single institution, and did not include other disciplines such as natural sciences or fine arts. Future studies should increase the sample to include more disciplines, types of institutions and resource contexts to enhance the robustness of the theoretical model.

## 5 Conclusion

This grounded theory study aimed to explore how non-verbal behaviors facilitate the identity transition from compliant learners to collaborative partners in online collaborative learning and tested the hypothesis that a three-stage mechanism of emotional resonance, boundary dissolution, and relational empowerment supports this transition. The findings support this hypothesis. Here, some conclusions are drawn.

First, non-verbal behaviors in online collaborative learning are categorized into three types: emotional expression, relationship coordination, and task support. Emotional expression refers to the use of emojis and interjections to express emotion and act as a bridge for emotional communication and the pursuit of emotional intimacy. Relationship coordination refers to synchronizing response rhythms and adapting interaction frequency, supporting relationship maintenance. Task-support behaviors consist of providing encouragement through document annotations and synchronizing co-editing steps, which empower co-creation. Together, these three behavioral categories play distinct roles in the identity transition process, forming a comprehensive system of symbolic interaction.

Second, non-verbal behaviors drive the identity transition of learners from compliant learners to collaborative partners through a progressive mechanism: “establishing a foundation through emotional resonance; breaking through frameworks by dissolving boundaries; confirming identity through relational empowerment.” Emotional expression behaviors lower identity barriers in online collaborative learning contexts by conveying goodwill and recognition, thereby eliciting emotional resonance. Relationship coordination behaviors dissolve the “supervisor-supervisee” classroom framework by aligning interaction rhythms and frequencies, thus loosening the role of the “compliant learner.” Task support behaviors empower learners with voice and decision-making authority through encouraging annotations and synchronous editing, thereby reinforcing the identity of “collaborative partners.”

Third, non-verbal symbols serve as the core vehicle for identity transformation in digital educational contexts. This study examines non-verbal symbols in online collaborative learning and demonstrates that identity is not an inherent individual attribute but rather a dynamic process continuously negotiated through symbols during interaction. As implicit carriers of symbols, non-verbal behaviors become the key resources for breaking through the rigidity of classroom roles and facilitating identity transformation.

## Data Availability

The original contributions presented in this study are included in this article/supplementary material, further inquiries can be directed to the corresponding author.

## Appendix A

Q1. In synchronous online classes, what are your typical non-verbal participation behaviors that align with instructional expectations? How do these behaviors reflect your institutional role as a student?

Q2. When transitioning from a classroom setting to a group collaboration task, what is the first non-verbal behavior you use to signal this shift in context?

Q3. Please recall a specific moment during group collaboration when a particular non-verbal interaction - such as an emoji, a tone of voice, or a simultaneous edit made you strongly feel a sense of team cohesion and partnership. Please describe the event in detail.

Q4. During collaboration, do other members’ response delays, interaction frequency, or the pace of task hand-offs affect your role positioning within collaborative learning?

Q5. What role do non-verbal behaviors, such as facial expressions and intonation—play in constructing and regulating the group’s emotional atmosphere and building interpersonal trust?

Q6. Have there been moments when you broke out of the passive pattern of merely responding to instructions by proactively initiating discussions, adjusting the pace of interaction, or changing your approach to task participation?

Q7. When you contributed ideas through text annotations, suggestive questions, or direct editing of shared documents, how did you perceive changes in your level of influence and decision-making participation within the group?

Q8. Looking back at the entire collaboration process, which non-verbal behaviors played an important role in your ultimate self-identification as a team collaborator? Please explain the core characteristics of these behaviors that facilitated this identity formation.
